# Multiscale Functional Connectivity analysis of episodic memory reconstruction

**DOI:** 10.3389/fcogn.2024.1433234

**Published:** 2024-07-31

**Authors:** Manuel Morante, Kristian Frølich, Muhammad Shahzaib, Sadia Shakil, Naveed ur Rehman

**Affiliations:** ^1^Department of Electrical and Computer Engineering, Aarhus University, Aarhus, Denmark; ^2^Department of Electrical Engineering, Institute of Space Technology, Islamabad, Pakistan; ^3^Department of Biomedical Engineering, The Chinese University of Hong Kong, Hong Kong, China

**Keywords:** Multivariate Variational Mode Decomposition (MVMD), fMRI, Default Mode Network (DMN), temporal pole, episodic memory (EM)

## Abstract

Our ability to share memories constitutes a social foundation of our world. When exposed to another person's memory, individuals can mentally reconstruct the events described, even if they were not present during the related events. However, the extent to which the neuronal connectivity patterns elicited by the mental reconstruction of an event mirror those present in the brains of individuals who experienced the original event remains unclear. Through two independent fMRI experiments, we explore the Functional Connectivity (FC) patterns at different timescales associated with these cognitive processes using the innovative Multiscale Functional Connectivity (MFC) technique. This study aims to shed light on how our brains construct mental representations of scenes in a movie compared to the verbal transmission of the same scenes. Our results demonstrated that the Default Mode Network (DMN) plays a crucial role in these experiments and exhibits unique FC patterns across different timescales, yet remarkably consistent among participants. In addition, we found significant connectivity patterns within the temporal cortex, including significant contributions of the temporal pole and the fusiform gyrus, which exhibited a pivotal role in cooperation with the DMN in both experiments.

## 1 Introduction

Humans can mentally reconstruct events when exposed to another person's memory, even if they were not physically present during the related events. Although this seemingly effortless skill is inherently private, personal, and prone to errors (Carpenter et al., 2021), it is also vital for sustaining our human societies (Wang, 2021). By sharing memories, we can empathize and form strong bonds with others. In turn, this helps us to build relationships, foster a sense of cultural identity, and comprehend our past (Schacter and Coyle, 1995). At the individual level, episodic memories shape our identity, drive how we set our goals, and help us make decisions (Schacter and Coyle, 1995). Furthermore, they play a fundamental role in several neurological disorders (Dere et al., 2010). For instance, the deficit of episodic memories stands as a recognized cognitive feature associated with depression (James et al., 2021).

These reasons make exploring how we form and transmit episodic memories an area of great interest, which bears profound implications for neuroscience, psychology, sociology, and even artificial intelligence (Martin et al., 2022). Understanding how memory works is relevant for gaining a better knowledge of human behavior and, at the same time, for advancing the research for developing effective neurological treatments. Nevertheless, studying the neurophysiological mechanisms that underlie episodic memories is challenging in practice. The human brain is a complex dynamic system constantly fluctuating at distinctive timescales, making analyzing brain signals profoundly challenging (Bolton et al., 2020).

For decades, researchers have used several neuroimaging techniques to investigate the human brain. One commonly used neuroimaging technique for this purpose is functional Magnetic Resonance Imaging (fMRI), which allows researchers to detect changes in brain activity by indirectly measuring the Blood Oxygen Level Dependent (BOLD) effect (Power et al., 2011; Morante et al., 2021). By analyzing fMRI data, researchers can infer the underlying Functional Connectivity (FC) patterns associated with the brain activity elicited during the scanner session.

Among other research areas of interest, linguist communication (Dronkers et al., 2007; Geranmayeh et al., 2014; Novén et al., 2021), and episodic memory (Fuster, 2009; Yoo et al., 2021) have been widely studied using fMRI techniques. Nonetheless, Zadbood et al. (2017) stands as the first fMRI study that simultaneously studied the full communication circle, i.e., how episodic memories are verbally transmitted and whether the active neuronal networks involved are similar to the activation patterns that appear when experiencing the same original event. To this aim, the authors studied how real-life episodic memories are encoded and transmitted to naive listeners using fMRI data. These results evidenced that similar neuronal activation patterns appear consistently during the encoding and reconstructing of an episodic event.

Conventional methods for analyzing fMRI data encounter several limitations that hinder the analysis and interpretation of brain activity (Bolton et al., 2020; Lurie et al., 2020). For example, even after preprocessing, fMRI data may contain significant information beyond neuronal activity patterns (Bianciardi et al., 2009), some of which resemble brain activity. Furthermore, it remains unclear if current preprocessing strategies accurately remove physiological cofounds, given their insidious similarity to neuronal response, as recently discussed by Chen et al. (2020). Moreover, accumulating evidence in recent years has revealed that the actual activation patterns within the brain present complex dynamic behavior at different timescales (Bolton et al., 2020), eliciting complex spatio-temporal patters (Ge et al., 2019; Iraji et al., 2020), as well as nonlinear (Morioka et al., 2020) and nonstationary neuronal dynamics.

Recently, Morante et al. (2024) proposed an alternative approach for extracting FC information from fMRI data referred to as Multiscale Functional Connectivity (MFC). This novel approach relies on a signal mode decomposition algorithm known as Multivariate Variational Mode Decomposition (MVMD) proposed by Rehman and Aftab (2019), which decomposes fMRI data in terms of their inherent oscillations, referred to as intrinsic modes (IMs) (Yuen et al., 2019; Morante et al., 2024). This process occurs in a data-driven way without user-defined filters or alternative inaccurate preprocessing steps. Then, we can use those intrinsic modes to uncover the FC at different timescales, providing a multiscale representation of fMRI activity. This novel approach contrasts with other conventional FC alternatives, which perform a static evaluation of the FC or try to extract relevant changes in the fMRI dynamics through specific dynamic models, e.g., using a sliding window (Lurie et al., 2020).

In this study, we built upon the study conducted by Zadbood et al. (2017), who investigated the underlying neuronal activation patterns elicited during encoding and transmission of episodic memories. In line with Zadbood et al. (2017), we aim to explore the hypothesis that the same neuronal activation patterns underlie the encoding and reconstruction of a given event. However, in this original study, we go a step further by examining the FC patterns using MFC, a novel approach introduced by Morante et al. (2024) that allowed us to analyze the FC patterns at different timescales. Our findings demonstrated that the Default Mode Network (DMN) plays a critical role in episodic memory, exhibiting distinct FC patterns across different timescales. In addition, we found significant connectivity patterns within the temporal cortex, including significant contributions of the temporal pole and the fusiform gyrus, which exhibited a pivotal role in cooperation with the DMN in both experiments. Through comparisons among experiments, our results indicate significant similarities between the connectivity patterns involved in memory encoding and event construction with remarkable consistency among individuals.

## 2 Materials and methods

### 2.1 Experiment description and fMRI data

In this study, we further investigate the transmission of episodic memories utilizing fMRI data. To this end, we leveraged the same fMRI experiment from Zadbood et al. (2017). In this section, we provide a concise overview of their study, including a brief description of the primary experiment and the essential details regarding the fMRI data.

Put succinctly, Zadbood et al. (2017) conducted an fMRI experiment to investigate how neural patterns associated with viewing specific scenes in a movie are encoded, recalled, and transferred to others via verbal communication. Their goal was to understand the extent to which the neural patterns elicited by mental construction in listeners resemble those found in the brains of the persons who experienced the original events.

As detailed by Zadbood et al. (2017), the experiment involved three main stages, covering the full communication circle: watching, recalling, and listening. In the watching stage, they selected a group of participants to watch a movie (watchers), while undergoing fMRI scanning. Separately, another participant watched the movie (speaker) and was instructed to recall the movie inside the scanner without any external cues. This spoken recall was recorded as an audio file. Finally, another group of participants (listeners), who had never seen the movie, listened to the audio recording of the speaker's verbal recall while undergoing an fMRI scanner. The result was two different fMRI groups: one group with individuals who watched the movie (watching group) and a second group with participants who listened only to the verbal recollection (listening group).

To ensure the robustness, Zadbood et al. (2017) replicated the experiment using two separate movies. Specifically, they utilized excerpts from the first episodes of the BBC television series Sherlock (24 minutes) and Merlin (28 minutes). Our study adopted the same methodology; we used the Sherlock movie for the parameter selection and general evaluation of the fMRI data, and then , once completed the study of Sherlock, we replicated the results from the Merlin movie.

Similarly to Zadbood et al. (2017), we examined the cognitive processes involved in experiencing a new episodic event; “watching” a movie and subsequently recalling/reconstructing the same event based on a verbal description provided by the speaker, i.e., “listening” to the speaker's recollection of the movie. Our goal for this study was to further explore and expand the results from Zadbood et al. (2017) using MFC, to examine the FC patterns elicited during episodic memory encoding and reconstruction at different time scales. Finally, we identified significant activation patterns associated with episodic memory encoding and reconstruction, by comparing those patterns with the FC elicited at rest, following a similar procedure as described by Romanello et al. (2022).

#### Selected networks and regions of interest

For this study, we used Glasser brain atlas (Glasser et al., 2016), a cortical multimodal-based brain atlas that divides the brain into 360 regions of interest (ROIs). While the Glasser atlas includes numerous cortical areas, not all are equally relevant to the experiments under study. Therefore, we focused on networks that play a relevant or critical role in episodic memory processing. Therefore, we selected ROIs based on this criterion, guided by the findings from the original experiment conducted by Zadbood et al. (2017) and related literature.

Firstly, among all of them, the Default Mode Network (DMN) holds particular interest, given the findings of Zadbood et al. (2017) and the nature of our experiments. Similarly, although it was beyond the primary scope of their original study, Zadbood et al. (2017) also provided evidence of substantial contributions to the Temporal Cortex (TpC). For instance, the authors demonstrated significant activity in the Fusiform Gyrus, which plays a role in face and emotion recognition (Chatzichristos et al., 2020). Moreover, we selected other critical areas within the temporal cortex, such as the auditory cortex and the temporal pole, e.g., Yoo et al. (2021); Wen et al. (2019), and the visual areas within the occipital cortex. Additionally, we incorporated some of the most common areas related to language and memory-related areas (Tobyne et al., 2017). These selections yielded 68 ROIs out of the 360 in the Glasser atlas. Table 1 summarizes the selected ROIs and their corresponding main network organization.

**Table 1:** Selected ROIs from the Glasser atlas and their main corresponding network of interest.

**Index**	**Label**	**Network**	
30	Area 7m	DMN	Default Mode Network
33	Area ventral 23 a+b		
34	Area dorsal 23 a+b		
61	Area a24		
62	Area dorsal 32		
64	Area p32		
65	Area 10r		
69	Area 9 middle		
72	Area 10d		
149	Area PFm complex		
150	Area PGi		
161	Area 31pd		
162	Area 31a		
74	Area 44	Lng	Language
75	Area 45		
80	Area IFJp		
81	Area IFSp		
129	Area STSd posterior		
18	Fusiform face complex	M&E	Memory and emotion processing
111	Anterior ventral insular area		
120	Hippocampus		
124	ParaBelt complex		
136	Area TE2 posterior		
8	Primary motor cortex	SMN	Somatomotor network
9	Primary sensory cortex		
12	Area 55b		
51	Area 1		
54	Dorsal area 6		
55	Area 6mp		
130	Area STSv posterior	TpC	Temporal cortex
131	Area TG dorsal		
172	Area TG ventral		
177	Area TE1 middle		
1	Primary visual cortex	Vis	Primary visual cortex

For simplicity, the table only shows the labels associated with the left hemisphere. The study included the ROIs from both hemispheres, rendering 68 ROIs.

#### fMRI data and preprocessing

We utilized the same dataset collected by Zadbood et al. (2017), which is freely available on OpenNeuro^1^. The dataset comprises 36 participants aged 18 to 45, all right-handed native English speakers with normal or corrected-to-normal vision. Participants were randomly assigned to watch either Sherlock (*n* = 18) or Merlin (n = 18). The fMRI data were acquired using a 3T scanner with a repetition time (TR) of 1500 ms, with detailed acquisition and processing parameters reported by Zadbood et al. (2017).

Preprocessing was conducted following a standard preprocessing pipeline, including slice timing correction, followed by corregistration and spatial normalization to the MNI space. We also performed motion correction followed by spatial smoothing of each volume with a 4-mm FWHM Gaussian kernel. For ROI extraction, we used the Glasser atlas. All these steps were conducted using AFNI toolbox ^2^.

In addition, we employed resting-state data from the WU-Minn Human Connectome Project (HCP) (Van Essen et al., 2013) to obtain significant connectivity patterns from the task-related experiments of interest. Specifically, we randomly selected 20 healthy participants (9 females, aged 22–35 years) from the HCP repository. The fMRI data were collected using a 3T scanner with a repetition time (TR) of 720 ms, with acquisition parameters detailed in the HCP imaging protocols^3^. For this study, we used the dataset already preprocessed, which includes some minimal preprocessing steps and normalization over the MNI space. In addition to the standard preprocessing steps conducted by the HCP, we performed spatial smoothing of each volume with a 4-mm FWHM Gaussian kernel. For the additional spatial smoothing and ROI extraction, we used the Nilearn toolbox .^4^

### 2.2 Overview of multiscale functional connectivity

Multiscale Functional Connectivity (MFC) is a novel approach for analyzing FC in fMRI, recently introduced by Morante et al. (2024). This technique extracts FC patterns among different timescales while, at the same time, it separates them from other interfering components.

The core idea behind MFC is to use MVMD^5^ to extract the natural oscillations in fMRI data associated with neuronal activity from different timescales. In summary, MVMD is a fully data-driven algorithm that decomposes a multivariate signal in terms of its natural intrinsic oscillations. Those intrinsic oscillations, often referred to as intrinsic mode functions in the literature (Dragomiretskiy and Zosso, 2014), consist of a particular family of amplitude- and frequency-modulated functions with a well-defined instant frequency at any given time instance (Huang et al., 1998; Dragomiretskiy and Zosso, 2014). In other words, those natural oscillations behave similarly to modulated harmonics that remain relatively close to a particular frequency (Daubechies et al., 2011). Yet, they are flexible enough to accommodate nonlinear and nonstationary fluctuations in the data (Dragomiretskiy and Zosso, 2014).

Similarly, MVMD offers several advantages compared to alternative signal mode decomposition approaches as discussed by Morante et al. (2024). Firstly, MVMD offers an entirely data-driven approach to decomposing fMRI data in terms of their inherent oscillations, alleviating the need to apply fixed and supervised preprocessing steps to the fMRI data. Second, MVMD is a multivariate algorithm that matches frequency content obtained from multiple brain areas, enabling information matching across multiple spatial and temporal scales. Then, as described by Morante et al. (2024), we can use these IMs to separate neurophysiological brain activity from other interfering components. We can do this by analyzing the central frequencies associated with each mode. Finally, we can use the time signals related to each mode to obtain their corresponding FC activation patterns. In summary, MFC exploits the data-driven nature of MVMD to unveil the natural IMs present in fMRI and then obtain their corresponding FC patterns.

#### Natural frequency components of fMRI data

Understanding the frequency organization of the fMRI signal and brain activity dynamics is crucial for MFC. While studies that focus on frequency-related aspects of fMRI are relatively rare, primarily due to the low temporal resolution inherent to fMRI data, existing research offers valuable insight into the frequency organization of the fMRI signal and brain activity dynamics. For instance, Cordes et al. (2001) demonstrates that the frequency contribution to the correlation patterns spans across various frequency bands. Similarly, Yuen et al. (2019) investigated the inherent frequency components across different brain locations, yielding similar findings.

Nonetheless, unlike other commonly used alternative neuroimaging techniques such as Electroencephalography (EEG) or Magnetoencephalography (MEG) (Koshev et al., 2021), in fMRI, neuronal activity is indirectly measured through the Blood Oxygen Level Dependent (BOLD) effect (Power et al., 2011), which limits the observable neuronal activation dynamics of the brain to lower frequencies (Preti et al., 2017). Similarly, the relatively low sampling ratio utilized by fMRI scanners also imposes a challenge when studying the frequency contributions to fMRI signals (Power et al., 2011), as it restricts the maximum potential accessible frequencies and, at the same time, can introduce aliasing with physiological components.

Despite all the limitations discussed above, fMRI frequency components comprise a rich spectrum that covers several relevant frequency bands. First, very low-frequency oscillations, lower than 10 mHz (Power et al., 2011), correspond to trends, scanner instabilities, and motion residuals. Neurophysiological activation patterns resulting from neuronal activity appear within the range of 10–200 mHz, e.g., Yuen et al. (2019) and Cordes et al. (2001) emphasizing the significant contribution of this frequency band to fluctuations related to brain activity, which correspond with the natural band dominated by the BOLD response.

Additionally, fundamental respiratory oscillations usually occur around 250 mHz, while the first harmonic of respiration appears around 500 mHz. Contributions from blood vessels and cerebrospinal fluid pulsations fall within 400 to 800 mHz. They also noted their relevance to correlations between several brain areas and even aliasing with other frequencies. Those high-frequency interfering frequencies are primarily driven by cardiac pulsations, and their effects can introduce significant changes in the lower bands due to aliasing (Cordes et al., 2001).

### 2.3 Data analysis and parameter selection

For each individual, we initially extracted the time series from the fMRI data using the Glasser atlas for the selected ROIs, as detailed in Table 1. Subsequently, we removed the average value among the ROIs. Following the extraction of the time series associated with the ROIs and adhering to the methodology proposed by Morante et al. (2024), we decomposed the time activations patterns among all the rows—in a multivariate fashion—using MVMD into their fundamental oscillatory components, i.e., their intrinsic modes.

We estimated the FC patterns associated with each IM. This process involved estimating Pearson's correlation among the selected ROIs over the modes within the natural frequency band, i.e., the one that corresponds to where neuronal-related activation patterns are anticipated. While it is feasible to examine FC among all the IMs (Morante et al. (2024)), we focused solely on those carrying neurophysiological information, namely, those whose main oscillatory component appears within the expected natural neurophysiological band.

#### Significant activation patterns

After obtaining the FC patterns associated with each IM, we performed several statistical tests to unveil relevant significant information. In particular, following the procedure reported by Romanello et al. (2022), we perform a statistical test using the obtained FC patterns from the different task-related experiments and the FC associated with the resting-state. We resorted to the use of resting-sate from the HCP because Zadbood et al. (2017) only conducted task-related experiments.

The goal is to unveil the activation patterns specific to the task of interest. To this aim, we first estimated the FC associated with resting-state data following the same procedure as described by Morante et al. (2024), who have already studied this data. Then, we aligned the central frequencies and obtained IMs associated with the resting state, and, finally, we assessed significant activation patterns using a permutation-based T-test corrected with a false discovery rate adjusted to *p* ≤ 0.05, as used by Romanello et al. (2022).

#### Parameter selection

Concerning the selection of the parameters for the MFC analysis and following the suggestions from Morante et al. (2024), only two parameters need to be selected: the number of intrinsic modes (*K*) and the parameter α from MVMD. Using the fMRI data only from the Sherlock experiment, we conducted a small parameter evaluation with varying values. We observed that the parameter α exhibited minimal sensitivity, and we set it to 1000 for simplicity. Furthermore, it was determined that setting the number of modes to *K* = 6 yielded optimal decomposition. For the resting state data from the HCP, we used the same parameter step up as described by Morante et al. (2024).

## 3 Results

This section presents the results of our study employing MFC, as delineated in Section 2. Consistent with the approach outlined by Morante et al. (2024), firstly, we assessed the frequency and energy distribution of the IMs. Then, we determined their corresponding FC patterns and evaluated their reliability among participants. Finally, after selecting the relevant neurological modes, we examined their correlation patterns and assessed significant activation patterns compared to the resting state fMRI data.

### 3.1 Intrinsic mode decomposition

#### Frequency and energy distribution

**Figure 1:**
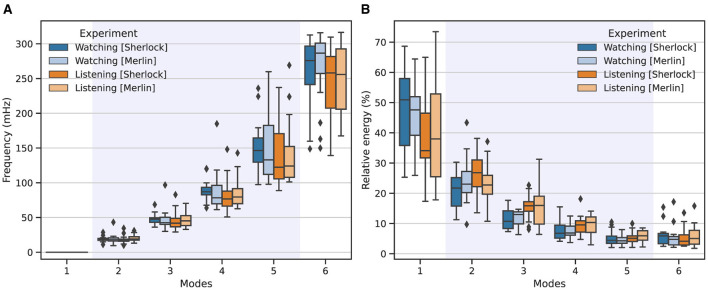
Frequency **(A)** and energy **(B)** distributions associated with each IM across the two studied experimental conditions: watching (blue) and listening (orange). Intense colors represent results from the Sherlock movie, while lighter colors denote those from the Merlin movie. The boxplots show the corresponding outcomes aggregated across all participants involved in the study, for *K* = 6 modes. The shadowed area highlights the modes that fell within the natural neurophysiological band.

Figure 1 shows the corresponding frequency and energy associated with each mode among all participants. Overall, we observed that high-energy modes corresponded to low-frequency (IMs 1, 2, 3), whereas modes with lower relative energy exhibited high-frequency (IMs 4, 5, 6). For instance, the first mode contains most of the signal's energy, which diminishes with increasing frequency.

Similarly, when comparing both experimental conditions, we observed great consistency. However, a closer inspection of the results revealed that modes 4 and 5 exhibited lower central frequencies than those associated with watching. Additionally, mode 1, for listening, exhibited consistently lower energy, whereas modes 2 and 3 showed consistently higher energy than the same modes associated with watching.

#### Reliability among participants

**Figure 2:**
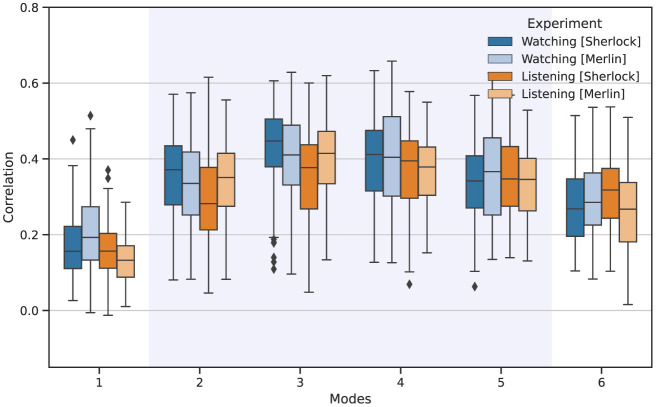
Reliability of the FC patterns associated with each IM among all participants for watching (blue) and listening (orange). The boxplots depict the Pearson correlation values obtained from all individual pair comparisons.

After obtaining the different IMs, we can determine the FC patterns associated with each mode, which encapsulates a distinct FC pattern. To verify the reliability and neurophysiological relevance of the modes, we systematically assessed similarity in terms of correlation between the FC patterns across all pairs of individuals. Figure 2 illustrates the similarity of the FC patterns obtained for each mode, displaying correlation values among FC for all the individual pair comparisons. Notably, the first and last modes exhibited the lowest similarity among individuals. In contrast, modes 3 and 4 consistently showed higher similarity.

### 3.2 Multiscale functional connectivity

After analyzing the frequency distribution and reliability of the different IMs, we concentrated on modes 2, 3, and 4. Although mode 5 fell within the potential neurophysiological range, we excluded it from the study because it may contain some interfering components or potential cofounds, as shown in the large variance in the central frequency shown in Figure 1 and its proximity to the maximum observable frequency.

**Figure 3:**
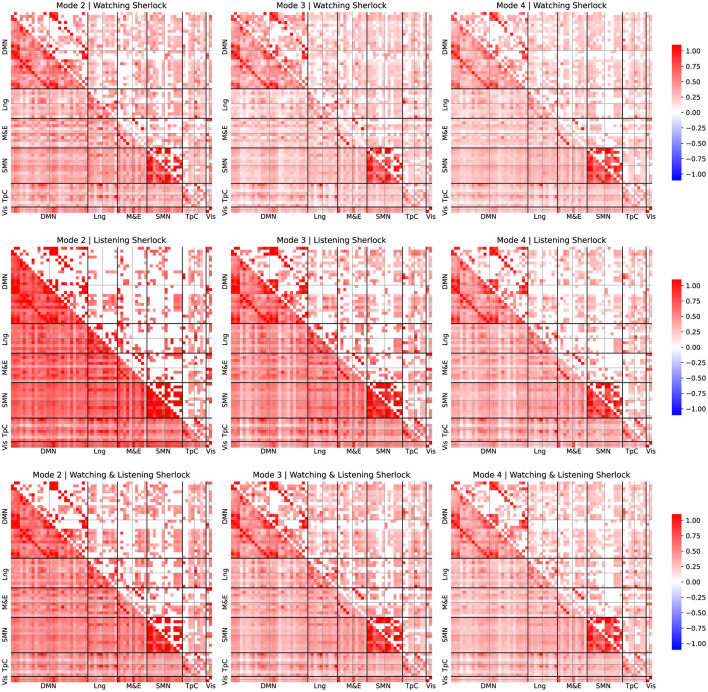
FC patterns of the modes 2, 3, and 4 and group comparison for Sherlock experiment. Mean static FC matrices were computed by averaging across all participants within each group. Pearson correlation coefficients were Fisher-Z transformed. The lower diagonal part shows the average correlation coefficients. The upper diagonal only displays the group comparison's significant activation coefficients with respect to the resting-state results from the relevant permutation-based *T*-test corrected with a false discovery rate adjusted to *p* ≤ 0.05.

**Figure 4:**
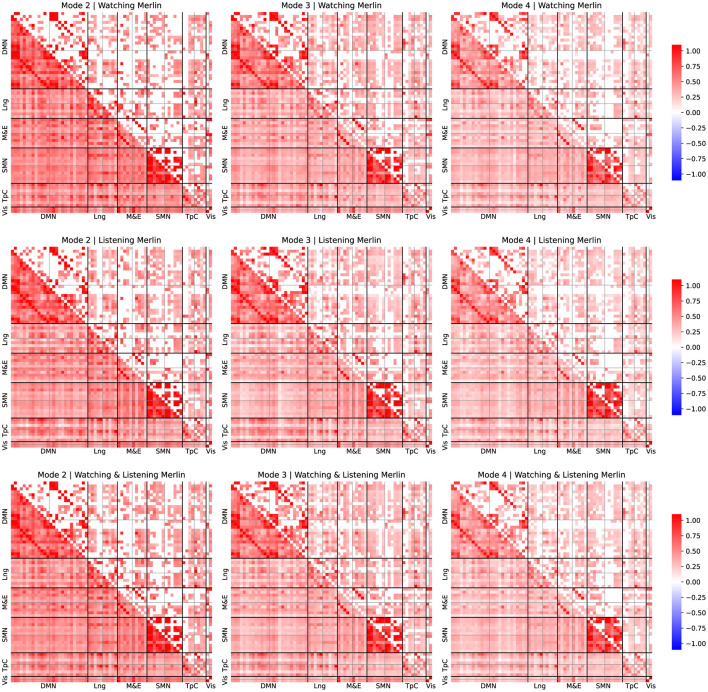
FC patterns of the modes 2, 3, and 4 and group comparison for Merlin experiment. Mean static FC matrices were computed by averaging across all participants within each group. Pearson correlation coefficients were Fisher-Z transformed. The lower diagonal part shows the average correlation coefficients. The upper diagonal only displays the group comparison's significant activation coefficients with respect to the resting-state results from the relevant permutation-based *T*-test corrected with a false discovery rate adjusted to *p* ≤ 0.05.

Figures 3, 4 depict average FC patterns for each mode. We obtained those FC maps by averaging the individual FC patterns across all participants in the Sherlock and Merlin experiments. Each column corresponds to a particular mode, and each row contains different comparisons: the first row refers to the watching experiment, the second corresponds to listening, and the last depicts the results common to watching and listening.

For all the comparisons, we performed a statistical test with respect to resting-state data to identify connectivity pattern that appeared significantly activated in contrast to the resting state, to reveal the FC patterns associated with the experimental tasks, following a similar approach as described by Romanello et al. (2022). Pearson's correlation coefficients were Fisher-Z transformed. The lower diagonal part displays average correlation coefficients, while the upper diagonal shows only significant activation coefficients compared to the resting-state results derived from permutation-based T-tests corrected with a false discovery rate adjusted to *p* ≤ 0.05.

For simplicity, we arrange the ROIs per leading network of interest from the left hemisphere in the first place and then from the right, following the order reported in Table 1. We examined these different networks individually to simplify the reporting process. Note that we focused on the results corresponding to the connectivity patterns common to both watching and listening for both studies since they are our study's primary goal.

#### Default Mode Network (DMN)

Upon examination of the results from Figures 3, 4, the connectivity within the DMN demonstrated a consistent correlation among all the obtained modes. However, we observed some significant differences between connectivity patterns among these modes.

The standard DMN connectivity pattern appeared to be separated into various modes. On the one hand, in mode 2, the medial prefrontal area exhibited significant connectivity with additional frontal areas and regions of the parietal cortex. In contrast, it lacked significant connectivity between the medial prefrontal regions and the ROIs within the precuneus. On the other hand, mode 4 displayed significant connectivity between the precuneus and medial frontal regions. However, it lacked the same connectivity patterns as mode 2 between the frontal and parietal cortex.

Furthermore, it is worth noticing that the connectivity patterns observed exhibited strong lateralization. Specifically, we observed that the right regions within the DMN appear more densely and consistently correlated among all the modes than the same areas in the left hemisphere.

#### Temporal cortex

We observed that the temporal cortex exhibits significant connectivity with some ROIs from the DMN. Mode 2 exhibited significant connectivity between the prefrontal cortex and the temporal pole, while modes 2 and 3 demonstrated notable connectivity between the temporal pole from both hemispheres and the angular gyrus. Mode 4 displayed fewer significant connectivity patterns, with the most relevant being between the medial and superior temporal cortex. Furthermore, these regions of the temporal cortex that exhibit significant connectivity with the DMN's ROIs are also found to be significantly correlated with language areas.

#### Memory and emotion regulation networks

We observed distinct behaviors related to episodic memory and emotion regulation networks across the different modes. Mode 2 and 3 displayed significant connectivity patterns between the prefrontal and somatomotor cortex and temporal areas. These areas included the fusiform gyrus, the temporal pole, and the precuneus. Mode 4 displayed fewer connections, with the most relevant in the temporal cortex.

Interestingly, we also observed a significant correlation between the fusiform area and the visual cortex among all the modes. Despite significant correlations in both hemispheres, the left fusiform area displayed a dominating role.

#### Language

We observed that most areas commonly associated with language significantly contribute to observed connectivity patterns. For instance, mode 4 exhibited significant connectivity between the medial temporal and inferior frontal areas within the right hemisphere. Also, mode 2 displayed significant connectivity within the inferior frontal regions, exhibiting lateralization. Furthermore, our analysis revealed that the connectivity patterns also consistently involved Broca's area, which showed strong correlations with the frontal regions of the default mode network (DMN). Interestingly, Broca's area exhibited a significant lateralization.

#### Somatomotor network (SMN)

Finally, we observed significant correlations within the somatosensory cortex in modes 2, 3, and 4. Mode 2 displayed a distinctive connection between the right and left regions within the DMN and exhibited strong lateralization, when observing the ROIs associated with the language area. However, these patterns were not present in modes 3 and 4, which appeared more homogeneous in terms of connectivity within the somatosensory cortex.

## 4 Discussion

Our study further investigated the mechanisms underlying episodic memory transmission. Specifically, we focused on analyzing the connectivity patterns of brain networks associated with processing episodic memories. We assessed whether similar neuronal mechanisms underlie the encoding and reconstruction of a given scene. To this end, we used the novel MFC technique proposed by Morante et al. (2024), which allowed us to explore the encoding of functional connectivity patterns at various timescales.

Regarding the MFC performance, our results are in line with the behavior reported by Morante et al. (2024); the energy and frequency distribution match the expected behavior and bandwidth for this experimental setup. Further scrutiny of Figure 1 allowed us to determine the neurophysiological relevance of each mode. Specifically, modes 2, 3, and 4 fell within the expected frequency range associated with natural neuronal activations (Cordes et al., 2001), which appeared centered around 19, 47, and 85 mHz. Conversely, mode 1 only contained trends and low-frequency residuals unrelated to neuronal activation, and mode 6, whose average frequency appeared around 254 mHz, is likely to contain respiration and other high-frequency interfering components. Mode 5, which has an average central frequency of 145 mHz, constitutes a particular case; although it exhibited a central frequency within the natural neurophysiological band, the high TR of this experiment may introduce additional interfering components, which seems to be reflected in the large variance of the central frequency associated with this IM in Figure 1. Therefore, although it may contain relevant information, we excluded it from our FC analysis to avoid potential bias.

The comparison of the FC patterns associated with each mode revealed notable consistency among participants for the IMs that carry the most significant neurophysiological information, as shown in Figure 2. Similarly, despite the relatively low number of participants, the consistent results among participants and the two different movies support the validity of MVMD for extracting functional connectivity patterns among individuals and are in line with the expected behavior reported in Morante et al. (2024). Of particular interest is mode 3, which emerged as the most consistent across the experimental conditions studied.

Conversely, mode 1 displayed the lowest correlation consistency among participants but the highest relative energy. The reason for this result is that mode 1 mainly contained trends, motion residuals, and other low-frequency drifts that affect the whole brain, yet carry no neurophysiological information. This observation also complies with the expected behavior for this mode as reported by Morante et al. (2024). Mode 6, which lies outside the bandwidth of interest, also showed low consistency among participants. However, it exhibited higher consistency among individuals than the first mode. As detailed in Morante et al. (2024), the reason is the presence of high-frequency structural noise from other physiological signals, e.g., heart-beat changes and respiratory variations, which often mimic neurophysiological signals and are relatively common among participants (Chen et al., 2020).

The analysis of the corresponding FC patterns associated with the neurophysiological IMs (see Figures 3, 4) revealed some interesting results. Firstly, we observed that the DMN played a crucial role in these experiments and exhibited distinct FC patterns across different timescales, yet is remarkably consistent across participants. This finding is consistent with the results reported in the original study by Zadbood et al. (2017), who highlighted the role of the DMN. However, unlike the original study, the MFC allowed us to further unveil the patterns of connectivity at different timescales within the DMN, as well as the participation of other relevant networks .

As depicted in Figures 3, 4, we observed that each mode reflected a distinct contribution to overall DMN activation. For instance, mode 2, which contains low-frequency responses, exhibited significant activity between the frontal and parietal areas of the DMN. Still, no significant correlations appeared between the frontal regions and the precuneus in this mode. Conversely, mode 4 showed significant connections between the frontal regions and the precuneus. As shown in Figure 1, we observed that the mean frequency associated with mode 4 is considerably larger than that of mode 2. Therefore, frontal and precuneus activity is more dynamic and strongly correlated as it is dominantly correlated at high frequencies. In contrast, the parietal areas of the DMN are unrelated to the precuneus as they primarily correlate with frontal regions at lower frequencies.

As briefly explained in Section 4, areas within the temporal cortex should also play a critical role and actively engage with the DMN (Tobyne et al., 2017; Wen et al., 2019). Interestingly, our results support this evidence. For instance, mode 2 showed connectivity within the auditory cortex and frontal areas, reflecting the participant's engagement with auditory stimuli. Mode 3 revealed correlations between the fusiform gyrus, precuneus, and temporal pole, possibly indicating retrieval of relevant content, imagination, and emotional processing. These results suggest that, in addition to the DMN, the temporal cortex play a crucial role in cooperating with the DMN in both experiments, including a significant contribution from the temporal pole and the fusiform gyrus.

The correlation between the fusiform area and the visual cortex suggests that the fusiform area is involved in visual processing, particularly in the recognition and perception of faces and other complex visual stimuli (Chatzichristos et al., 2020). This finding highlights the importance of the fusiform area in our ability to identify and distinguish visual information. Moreover, the connectivity between the temporal cortex and the angular gyrus suggests a potential involvement of these brain regions in language processing and semantic integration.

Additionally, our analysis revealed significant correlations between the language-related and temporal cortex and some memory and emotional processing areas, including the hippocampus. These findings suggest that the temporal cortex, along with the hippocampus, plays a crucial role in memory processing and emotional experiences (Bird and Burgess, 2008). The correlations between language-related regions and memory areas further support the idea that language and memory are closely intertwined (Yoo et al., 2021).

Modes 2 and 3 showed significant correlations between the temporal pole and several ROIs of the DMN, suggesting that the temporal pole and the DMN work collaboratively. As shown in Figures 3, 4, we observed similar patterns in both watching and listening tasks (as well as in the joint analysis), which suggests that the same neural networks actively participate in both tasks, despite their entirely different nature, which further supports the hypothesis that the same networks can engage in similar yet different tasks.

Finally, we want to emphasize that MFC has enabled us to gain a more comprehensive understanding of the different dynamics interactions within the DMN and their interactions with other networks at several timescales. As detailed above, MFC has been the key to studying the interactions of several networks at different frequencies, and it highlights the complex nature of memory encoding and retrieval of episodic memories.

###  Limitations and future work

This fMRI study focused on investigating the multiscale FC of two different fMRI experiments associated with the reconstruction of episodic memories. However, as with any experiment, there are some limitations.

Firstly, the number of participants is relatively small. We acknowledge that this limited sample size can affect the FC study, compromise the robustness of statistical tests performed in this study, and may hinder the generality of some of these findings. Nonetheless, the excellent consistency of the FC among participants indicates that the observed results generalize well among all individuals (see Figure 2). Of course, conducting further experiments with additional participants may enhance the reported results of this study.

Secondly, this study explored two specific scenarios within the information cycle, i.e., watching and listening to two different movies. As also acknowledged by Zadbood et al. (2017), how these findings may generalize to other real-world situations remains unclear. Similarly, further exploration of other episodic memory reconstruction-related scenarios could also benefit the understanding of how we transmit episodic memories.

Finally, data were collected using a 3T scanner, which provides higher TR than more advanced scanners with higher static magnetic fields. This posed a substantial limitation, restricting the accessible frequencies during the analysis and the number of modes that we could safely explore, e.g., mode 5. Therefore, data collected with a higher static magnetic field scanner may offer further insights; see, for example Morante et al. (2024).

## 5 Conclusions

In this study, we provide an overview and extend the findings by Zadbood et al. (2017), shedding light on how our brain processes episodic memories. Through the decomposition of fMRI activity into distinct intrinsic modes using MFC, we unveiled interesting connectivity patterns in several neuronal networks across different timescales. Overall, the study of the connectivity patterns associated with neurophysiological modes exhibited remarkable consistency among all studied participants and provided further evidence supporting the hypothesis that similar neuronal patterns underlie both episodic memory encoding and reconstruction. Consistent with prior research, our results further highlight the role of the DMN, while uncovering its dynamic nature among different timescales. Additionally, they also shed light on the intertwined role of the temporal cortex, particularly the temporal pole and the fusiform gyrus, which displayed a pivotal role in coordination with the DMN.

## Data availability statement

Publicly available datasets were analyzed in this study. This data can be found here: OpenNeuro and Human Connectome Project, https://openneuro.org/datasets/ds001110/versions/00003.

## Ethics statement

Ethical approval was not required for the study involving humans in accordance with the local legislation and institutional requirements. Written informed consent to participate in this study was not required from the participants or the participants' legal guardians/next of kin in accordance with the national legislation and the institutional requirements.

## Author contributions

MM: Conceptualization, Investigation, Methodology, Supervision, Validation, Visualization, Writing – original draft, Writing – review & editing. KF: Conceptualization, Data curation, Formal analysis, Investigation, Methodology, Resources, Software, Visualization, Writing – original draft, Writing – review & editing. MS: Data curation, Resources, Writing – review & editing. SS: Conceptualization, Methodology, Resources, Supervision, Writing – review & editing. NR: Conceptualization, Funding acquisition, Investigation, Methodology, Resources, Supervision, Validation, Writing – original draft, Writing – review & editing.

## Footnotes

^1^OpenNeuro dataset: https://openneuro.org/datasets/ds001110/versions/00003.

^2^AFNI: https://afni.nimh.nih.gov/.

^3^HCP 3T Imaging Protocol Overview: http://protocols.humanconnectome.org/HCP/3T/imaging-protocols.html.

^4^Nilearn: https://nilearn.github.io/stable/index.html.

^5^The code for MVMD is openly available on the MATLAB file exchange: https://se.mathworks.com/matlabcentral/fileexchange/72814-multivariate-variational-mode-decomposition-mvmd.
